# Dirac potential in a rotational dissipative quantum system

**DOI:** 10.1038/s41598-018-35763-z

**Published:** 2019-02-07

**Authors:** Yi-Rong Ma, Wei Jia, Shi-Rong Lin, Qing Zhao

**Affiliations:** 1 Center for Quantum Technology Research, School of Physics, Beijing Institute of Technology, Beijing, 100081 People’s Republic of China; 2 Southwest Institute of Technical Physics, Chengdu, 610041 People’s Republic of China

## Abstract

This study proposes the usage of an effective potential to investigate a dissipative quantum system with rotational velocity. After gauge transformation, a Doebner- Goldin equation (DGE) that describes the dissipative quantum system with a Dirac potential is obtained. The DGE is solved based on constraint of vertical relation between the rotational velocity field and density gradient when a harmonic oscillator model is considered. It is observed that the dissipative quantum system is directly equivalent to a monopole system and that the two gauge potentials that are given by Wu and Yang in the north and south hemispheres can be reproduced. Furthermore, a set of gauge-invariant parameters is obtained to discuss the dissipation characteristics of the system.

## Introduction

The Dirac potential has been introduced by Dirac as part of the discussion related to magnetic monopoles in 1931^1^. It possesses a string of singularity in the gauge potential field and provides a special concept to investigate the vector potential in quantum mechanics^2,3^. Based on this motivation, several theoretical studies were conducted^4,5^. Recently, Dirac monopole has been extensively investigated in exotic spin ices^6,7^, superfluid ^3^He^8,9^ and a Bose-Einstein condensate (BEC) system^10–13^. Furthermore, it was observed that the velocity potential could become completely equivalent to the Dirac potential in a spinor BEC system when the spinor order parameter was proposed^2,3^. A BEC without a spinor order is usually described using the Gross-Pitaevskii equation(GPE)^14,15^, and it is difficult to find the analogous Dirac potential by defining the velocity potential in such a system. However, the introduction of a spinor order parameter allows the GPE to become the extended GPE, and the velocity potential is observed to subsequently become equivalent to the Dirac potential. This indicates that the Dirac potential can be generated with considerable ease in a different quantum system that can be described using the nonlinear Schrödinger equation (NLSE) containing several nonlinear terms.

The DGE is one of the most general NLSEs and can be derived using the algebraic frame of group theory^16^. The DGE contains a set of nonlinear terms and can be used to describe dissipative quantum systems^17–21^. Furthermore, the equivalent Dirac potential may be obtained by defining the velocity potential in DGE. However, it is difficult to provide a clear explanation of the nonlinear terms in DGE because it is difficult to directly obtain an analytical solution of DGE.

To obtain an analytical solution of DGE and a better physical interpretation of the nonlinear terms, a dissipative quantum system with a rotational velocity was considered in the time-independent case. After the introduction of an effective potential, such as quantum pressure^14,22–24^, the resulting subfamily of DGE is observed to become similar to the analogous classical fluid equation. Based on this analogy, the simple three-dimensional DGE can be solved and a set of gauge-invariant parameters can be obtained when a central potential (such as the harmonic oscillator) and a constraint related to the vertical relation between the rotational velocity field and density gradient are suggested. Further, the gauge-invariant parameters characterize the physical properties of dissipation and exhibit that the Galilean invariance is broken^25,26^ in this dissipative system that is described using the subfamily of DGE. Additionally, the velocity potential and nonlinear terms of this system provide two gauge potentials *A*_*N*_ and *A*_*S*_^11^ in Case 1 and Case 2, respectively, which result from the DGE solution and process of gauge transformation.

This study is organized as follows. In Section 2, the Schrödinger equation is introduced to describe the motion of a charged particle that interacts with a rotational field. In Section 3, the DGE with Dirac potential is obtained. In Section 4, the analytical solution of the DGE and the physical meaning of the corresponding results are presented, whereas the dissipation characteristics of the system are discussed in Section 5. Finally, the main conclusions are presented in Section 6.

## The Model and Assumptions

The motion of a charged particle in an electromagnetic field is considered. The Schrödinger equation can be given as follows:

1 $$i\hslash \frac{\partial \psi ({\bf{r}},t)}{\partial t}=[\frac{1}{2\mu }{(\hat{P}-\frac{q}{c}{\bf{A}})}^{2}+V]\psi ({\bf{r}},t),$$

where *μ* is the mass of the particle, $\hat{P}=-\,i\hslash \nabla$ is the momentum operator, *c* is the speed of light, *q* is the charge, *ψ*(**r**, *t*) is the wave function, **A** is the vector potential, and *V* is the total potential that includes the dissipation of this system. For the time-dependent state, the wave function can be often expressed in an explicit form as $\psi ({\bf{r}},t)=\sqrt{\rho ({\bf{r}},t)}{e}^{i\zeta ({\bf{r}},t)}$, where *ρ*(**r**, *t*) is the density and the phase is *ζ*(**r**, *t*). Using the commutation relation between $\hat{P}$ and $\hat{{\bf{A}}}$, we obtain

2 $$[\hat{P},\hat{{\bf{A}}}]=-\,i\hslash \nabla \cdot {\bf{A}}$$

Further, Eq. (1) can be rewritten as the two following equations:

3 $$\frac{\partial \rho }{\partial t}+\nabla \cdot (\rho {\bf{v}})=0$$

4 $$\frac{\partial {\bf{v}}}{\partial t}+\nabla (\frac{1}{2}{{\bf{v}}}^{2})=-\,\frac{1}{\mu }\nabla (\frac{-\frac{{\hslash }^{2}}{2\mu }{\nabla }^{2}\varphi +V\varphi }{\varphi })-\frac{q}{c}\frac{\partial {\bf{A}}}{\partial t},$$

where $\varphi =\sqrt{\rho ({\bf{r}},t)}$ is the amplitude of wave function, ${\bf{v}}=\frac{1}{\mu }(\hslash \nabla \zeta -\frac{q}{c}{\bf{A}})$ is the velocity potential. Note that the corresponding vorticity is

5 $$\nabla \times {\bf{v}}=\nabla \times \frac{1}{\mu }(\hslash \nabla \zeta -\frac{q}{c}{\bf{A}})=-\,\frac{q}{\mu c}{\bf{B}}\ne 0.$$

Further, Eq. (4) can be rewritten as:

6 $$\frac{\partial {\bf{v}}}{\partial t}+({\bf{v}}\cdot \nabla ){\bf{v}}=-\frac{1}{\mu }\nabla (\frac{-\frac{{\hslash }^{2}}{2\mu }{\nabla }^{2}\varphi +V\varphi }{\varphi })-{\bf{v}}\times (\nabla \times {\bf{v}})-\frac{q}{c}\frac{\partial {\bf{A}}}{\partial t}\mathrm{.}$$

In the time-independent situation, by assuming that the direction of the density gradient is perpendicular to the direction of velocity and by considering

7 $$(-\frac{{\hslash }^{2}}{2\mu }{\nabla }^{2}+V-\mu h+\mu s)\varphi =E\varphi ,$$

where *s* satisfies ▽*s* = **v** × (▽ × **v**), *h* is a function that only depends on position and *E* is an eigenvalue of energy, the simplified form of Eq. (3) and Eq. (6) can be obtained as follows:

8 $$\nabla \cdot {\bf{v}}=\mathrm{0,}\,({\bf{v}}\cdot \nabla ){\bf{v}}=-\,\nabla h\mathrm{.}$$

It can be observed that the time-independent Schrödinger equation leads to the analogous classical fluid equations. The term *V*_*eff*_ = −*μh* + *μs* is defined as an effective potential term that is only related to velocity and can be considered to be the gauge potential. Such an introduction is equivalent to the gauge transformation ▽ → ▽ + **v** in Eq. (1), which is the original Schrödinger equation. Correspondingly, the wave function *ψ*(**r**) is also transformed into $\psi ^{\prime} ({\bf{r}}\text{'})=\psi {e}^{-\frac{i\mu }{\hslash }{\int }^{r}{\bf{v}}(r^{\prime} )dr^{\prime} }$ and renders Eq. (7) to be tenable if the phase vanishes.

To solve Eq. (8) in a simple manner, the solution of a similar set of equations that describe the steady-state flow of a classical fluid model is followed^27–29^. The velocity field of such a model depends only on the radius *r* and the angle *θ* in spherical coordinates:

9 $${v}_{r}=\frac{1}{r}\frac{{A}_{2}-{A}_{1}\,\cos \,\theta }{\sqrt{{A}_{1}\,{\cos }^{{\rm{2}}}\,\theta -2{A}_{2}\,\cos \,\theta -2{A}_{3}}},$$

10 $${v}_{\theta }=-\frac{\sqrt{{A}_{1}{\cos }^{{\rm{2}}}\,\theta -2{A}_{2}\,\cos \,\theta -2{A}_{3}}}{r\,\sin \,\theta },$$

11 $${v}_{\phi }=0,$$

where *A*_1_, *A*_2_, *A*_3_ are constants. The velocity is rotational ($\nabla \times v\ne 0$) when *A*_1_, *A*_2_, *A*_3_ become special in two cases:

Case 1: *A*_1_ = 0, *A*_2_ = − *A*_3_ = *D*^2^

12 $${{\bf{v}}}_{a}=\frac{1}{\sqrt{\mathrm{2(1}-\,\cos \,\theta )}}[-\frac{D}{r}\hat{r}+\frac{2D}{r\,\sin \,\theta }(1-\,\cos \,\theta )\hat{\theta }];$$

Case 2: *A*_1_ = 0, *A*_2_ = *A*_3_ = −*D*^2^

13 $${{\bf{v}}}_{b}=\frac{1}{\sqrt{\mathrm{2(1}+\,\cos \,\theta )}}[-\frac{D}{r}\hat{r}+\frac{2D}{r\,\sin \,\theta }\mathrm{(1}+\,\cos \,\theta )\hat{\theta }]\mathrm{.}$$

The corresponding effective potential *V*_*eff*_ = −*μh* + *μs* can be solved as follows:

14 $${V}_{a}=\frac{2\mu {D}^{2}\mathrm{(5}-3\,\cos \,\theta )}{5{r}^{2}{\sin }^{{\rm{2}}}\,\theta }$$

15 $${V}_{b}=\frac{2\mu {D}^{2}\mathrm{(5}+3\,\cos \,\theta )}{5{r}^{2}\,{\sin }^{2}\,\theta }$$

where *D* = *ℏ*/*μ*.

Substituting Eq. (14) and Eq. (15) into Eq. (7), the energy eigenvalue equation can be rewritten as

16 $$-\frac{{\hslash }^{2}}{2\mu }{\nabla }^{2}\varphi +V\varphi +[\frac{2\mu {D}^{2}\mathrm{(1}\mp \,\cos \,\theta )}{{r}^{2}\,{\sin }^{{\rm{2}}}\,\theta }\pm \frac{4\mu {D}^{2}\,\cos \,\theta }{5{r}^{2}\,{\sin }^{{\rm{2}}}\,\theta }]\varphi =E\varphi ,$$

where ∓ and ± in the third term are the signs for Case 1 and Case 2, respectively.

## The Dirac Potential and Dissipative Quantum System

The Hamiltonian of Eq. (7) can be rewritten as follows:

17 $$H=-\,\frac{{\hslash }^{2}}{2\mu }{\nabla }^{2}+\frac{2\mu {D}^{2}\mathrm{(1}\mp \,\cos \,\theta )}{{r}^{2}{\sin }^{2}\theta }+V^{\prime} ,$$

where the first term of the effective potential *V* in Eq. (14) and Eq. (15) is merged into the external potential and where $V^{\prime} =V\pm \frac{4\mu {D}^{2}\,\cos \,\theta }{5{r}^{2}{si}{{n}}^{2}\theta }$. By considering an equivalent relation between the monopole strength *g* = *ℏc*/*q* and *D* = *ℏ*/*μ* in Eq. (16), it can be observed that the Hamiltonian in Eq. (17) becomes similar to that of a particle’s motion in a monopole external field^30^:

18 $${H}_{M}=\frac{1}{2\mu }{(-i\hslash \nabla -\frac{q{{\bf{A}}}_{M}}{c})}^{2}+{V}_{M},$$

where the subscript *M* represents the monopole system, further, the external potential *V*_*M*_ = 0 is often considered in the monopole system. Here a relation *V*_*M*_ = *V*′ is required to calculate the density of the quantum system in Eq. (16). By expanding the first term of *H*_*M*_ and by temporarily ignoring other characteristic constants,

19 $${(-i\nabla -{{\bf{A}}}_{M})}^{2}=-\,{\nabla }^{2}+i\nabla \cdot {{\bf{A}}}_{M}+i{{\bf{A}}}_{M}\cdot \nabla +{{\bf{A}}}_{M}^{2}\mathrm{.}$$

The Dirac potential **A**_*M*_ in the study is divided into two regions with different values to eliminate the string singularity^11^:

20 $$\begin{array}{rcl}{R}_{a}:\,{{\bf{A}}}_{N} & = & \frac{g\mathrm{(1}-\,\cos \,\theta )}{rsin\theta }{\hat{e}}_{\varphi },\,\,\,\,0\le \theta  < \frac{\pi }{2}+\delta ;\\ {R}_{b}:\,{{\bf{A}}}_{S} & = & \frac{-g\mathrm{(1}+\,\cos \,\theta )}{rsin\theta }{\hat{e}}_{\varphi },\,\,\frac{\pi }{2}-\delta  < \theta \le \pi ,\end{array}$$

where *δ* was selected to satisfy 0 < *δ* ≤ *π*/2. The two gauge potentials can be transformed from one to the other using the gauge transformation relation

21 $${{\bf{A}}}_{N}={{\bf{A}}}_{S}+\frac{i}{q}{U}^{-1}\nabla U\mathrm{.}$$

Note that the Dirac potential **A**_*M*_(*θ*) is parallel to the ${\hat{e}}_{\varphi }$ direction while the wave function *ψ*_*M*_ is *ϕ*-independent^11^, so they satisfy ▽⋅**A**_*M*_ = 0 and **A**_*M*_⋅▽*ψ*_*M*_ = 0. After the second and third terms of Eq. (19) are eliminated, it can be observed that the sum of the effective potential and the external potential terms becomes equal to the square of the Dirac potential. Furthermore, **A**_*N*_ and **A**_*S*_ can be reproduced in two different cases using Eqs (12) and (13).

For the external potential *V*′, a modified nonlinear term is added along with the original scalar potential term *V*_0_. This nonlinear term is suggested as a summation of specific nonlinear terms in the following manner:

22 $$\frac{4\mu {D}^{2}\,\cos \,\theta }{5{r}^{2}\,{\sin }^{{\rm{2}}}\,\theta }={\rm{\Omega }}\{\varphi \mathrm{\}.}$$

A nonlinear term, such as Ω{*ϕ*}, is always introduced in the Schrödinger equation and models the quantum dissipation and diffusion effects. Further, Eq. (16) becomes equivalent to the general DGE^16,31,32^ in the time-independent state.

23 $$[-\frac{{\hslash }^{2}}{2\mu }{\nabla }^{2}+{V}_{0}+\frac{2\mu {D}^{2}\mathrm{(1}\mp \,\cos \,\theta )}{{r}^{2}\,{\sin }^{{\rm{2}}}\,\theta }\pm {\rm{\Omega }}\{\varphi \}]\varphi =E\varphi \mathrm{.}$$

As one of the most general NLSE, the DGE is always used to describe the dissipative quantum system and the dissipative term Ω{*ϕ*} can be written in terms of real and imaginary parts^25^

24 $${\rm{\Omega }}\{\varphi \}=R\{\varphi \}+iI\{\varphi \},$$

*R*{*ϕ*} and *I*{*ϕ*} are the real-valued nonlinear functions of the following form:

25 $$R\{\varphi \}=\hslash F^{\prime} \sum _{j=1}^{5}\,{c}_{j}{R}_{j}[\varphi ],\,I\{\varphi \}=\frac{\hslash }{2}F{R}_{2}[\varphi ],$$

where ${R}_{1}=(\nabla \cdot \hat{{\bf{j}}}/\rho )$. ${R}_{2}=({\nabla }^{2}\rho /\rho )$. ${R}_{3}={\hat{{\bf{j}}}}^{2}/{\rho }^{2}$.${R}_{4}=\hat{{\bf{j}}}\cdot \nabla \rho /\rho$, and ${R}_{5}={(\nabla \rho )}^{2}/{\rho }^{2}$ in which $\rho ={\varphi }^{\ast }\varphi$ and $\hat{{\bf{j}}}=\hslash ({\varphi }^{\ast }\nabla \varphi -\varphi \nabla {\varphi }^{\ast }\mathrm{)/2}\mu i$ denote the density and the current, respectively. *F* and *F*′ are the real-valued diffusion coefficients. Because of Eq. (22), we assume $F=0$ in this study^33^.

The potential of the quantum system was divided into three parts in this section. The first part is the original external potential *V*_0_, which requires a reasonable form to determine the density. The value of the second part can be observed in the second term of Eq. (23), and becomes equal to the square of the Dirac potential, which indicates that the model can be used to analogize the Dirac monopole system. The third part of the potential Ω{*ϕ*} describes the properties of the dissipative system. Therefore, the solution of Eq. (23) may provide a method to study both the Dirac monopole system and the diffusion system described in DGE.

## Analytical Result of DGE

For simplicity, let us consider *ϕ* = *R*(*r*)Θ(*θ*)*e*^*imϕ*^ and *ℏ* = 1. Because the fluid velocity is **v** = (*v*_*r*_, *v*_*θ*_, 0) without the component of $\hat{\phi }$, we obtain *m* = 0. By separating the variables, Eq. (16) can be reduced to:

26 $$[-\frac{1}{2\mu {r}^{2}}\frac{\partial }{\partial r}({r}^{2}\frac{\partial }{\partial r})+V^{\prime} ({\bf{r}})-E]R(r)=0$$

and

27 $$[-\frac{1}{\sin \,\theta }\frac{\partial }{\partial \theta }(\sin \,\theta \frac{\partial }{\partial \theta })+\frac{2\mu {D}^{2}\mathrm{(1}\mp \,\cos \,\theta )}{{r}^{2}{\sin }^{{\rm{2}}}\theta }]{\rm{\Theta }}(\theta )=\lambda {\rm{\Theta }}(\theta ),$$

where *λ* is a coefficient of variable separation.

First cosθ is rewritten as cosθ = *x*, and Eq. (27) becomes Heun’s differential equation^34^, which is similar to the equations related to the movement of a charged particle around a monopole in two regions. Because the two potentials in Eq. (14) and Eq. (15) were observed to be identical after gauge transformation, the equation in region *R*_*a*_ must be considered:

28 $$-\mathrm{(1}-{x}^{2})Y^{\prime\prime} +2xY^{\prime} +\frac{{(m+qx)}^{2}}{1-{x}^{2}}Y+{g}^{2}Y=\lambda Y\mathrm{.}$$

Because *Y*_*l*, *q*, 0_ is single valued, the gauge transformation relation between two regions requires *l* − *q* = *integer* and *l*(*l* + 1) ≥ *q*^2^. In this discussion, *q* = *m* = *D*, *α* = 0, *β* = −2*D*, and *n* = *l* + *q*. Further, solution *Y*_*l*, *q*, 0_ of Eq. (28) can be denoted as^33–35^

29 $${Y}_{l,q,0}={{\rm{\Theta }}}_{l,q}={2}^{D}{[\frac{2l+1}{4\pi }]}^{1/2}{\mathrm{(1}+x)}^{-D}{P}_{n}^{0,-2D}(x),$$

where

30 $${P}_{n}^{0,-2D}(x)=\frac{{(-\mathrm{1)}}^{n}}{{2}^{n}n!}{\mathrm{(1}+x)}^{2D}\frac{{d}^{n}}{d{x}^{n}}\mathrm{[(1}-x{)}^{n}{\mathrm{(1}+x)}^{n}\mathrm{].}$$

Note that the Dirac charge quantization condition is *q* = *l* = *N*/2, *N*∈${\mathbb{Z}}$ in this charge-monopole system with *V*_*M*_ = 0. However, the influence of the external potential *V*_*M*_ = *V*′ = *V*_0_ + Ω{*ϕ*} cannot be neglected, therefore, a reasonable scalar potential *V*_0_ must be suggested to modify the quantum number.

In general, if a potential satisfies *r*^2^*V*_0_(**r**) → 0 and the forms of the relevant wave function conform to *R*_*l*_(**r**)∝*r*^*l*^ when *r* → 0, the corresponding solution will properly satisfy the constraint condition of ▽*ρ*⋅**v** = 0. By considering the model of a three-dimensional isotropic harmonic oscillator, *V*_0_ can be expressed as follows:

31 $${V}_{0}(r)=\frac{1}{2}\mu {\omega }^{2}{r}^{2},$$

where *μ* is the mass of a single particle and *ω* is the angular frequency of a classical harmonic oscillator in the absence of an external force. The corresponding energy eigenvalues and solution of the Hamiltonian ${H}_{0}=-\,\frac{1}{2\mu {r}^{2}}\frac{\partial }{\partial r}({r}^{2}\frac{\partial }{\partial r})+{V}_{0}({\bf{r}})$ can be written as

32 $${E}_{{n}_{r},l^{\prime} }=2{n}_{r}+l^{\prime} +\frac{3}{2},\,\,{n}_{r},l^{\prime} =0,1,2,\cdots .$$

33 $$R(r)={\alpha }^{\mathrm{3/2}}{[\frac{{2}^{l^{\prime} +2-{n}_{r}}\mathrm{(2}l^{\prime} +2{n}_{r}+\mathrm{1)}}{{\pi }^{\mathrm{1/2}}{n}_{r}{\mathrm{[2}l^{\prime} +\mathrm{1]}}^{2}}]}^{\mathrm{1/2}}{(\alpha r)}^{l^{\prime} }{e}^{-{\alpha }^{2}{r}^{2}\mathrm{/2}}F(-{n}_{r},l^{\prime} +\frac{3}{2},{\alpha }^{2}{r}^{2}),$$

where $a=\sqrt{\mu \omega /\hslash }$, *F*(*α*, *γ*, *δ*) is the confluent hypergeometric function, *n*_*r*_ is the radial quantum number, and *l*′ the azimuthal quantum number that satisfies the modified relation of *l*′(*l*′ + 1) = *λ* − *g*^2^, where *g*^2^ = 3*D*^2^ − *D*. For the chosen model with a central potential, the relation between *l* and *q* can be easily obtained as

34 $$l^{\prime} =2q,\,\,\,\,l^{\prime} =0,1,2,\cdots \mathrm{.}$$

The energy is also quantized in this situation. Under the above constraints, the solutions of *R*(*r*) and Θ(*θ*) can be obtained as follows:

35 $${\rm{\Theta }}(\theta )={(-\mathrm{1)}}^{2q}\sqrt{\frac{2q+1}{4\pi }}{(\frac{1-\cos \theta }{2})}^{q}$$

and

36 $$R(r)={\alpha }^{\mathrm{3/2}}{[\frac{{2}^{2q+2}}{{\pi }^{\mathrm{1/2}}\mathrm{(4}q+\mathrm{1)!!}}]}^{\mathrm{1/2}}{(\alpha r)}^{2q}{e}^{-{\alpha }^{2}{r}^{2}\mathrm{/2}}.$$

Therefore, the probability density of a particle can be presented using Eq. (35) and Eq. (36).

37 $$\rho ={\varphi }^{2}={C}_{n,q}{\mathrm{(1}-\cos \theta )}^{2q}{r}^{4q},$$

where

38 $${C}_{{n}_{r},q}={\alpha }^{\mathrm{3/2}+2q}\sqrt{\frac{2q+1}{4\pi }}{[\frac{{2}^{2q+2-{n}_{r}}\mathrm{(4}q+2{n}_{r}+\mathrm{1)}}{{\pi }^{\mathrm{1/2}}{n}_{r}{\mathrm{[4}q+\mathrm{1]}}^{2}}]}^{\mathrm{1/2}}F(-{n}_{r},2q+\frac{3}{2},{\alpha }^{2}{r}^{2})$$

is a coefficient that changes with *n*_*r*_ and *q*.

**Figure 1:**
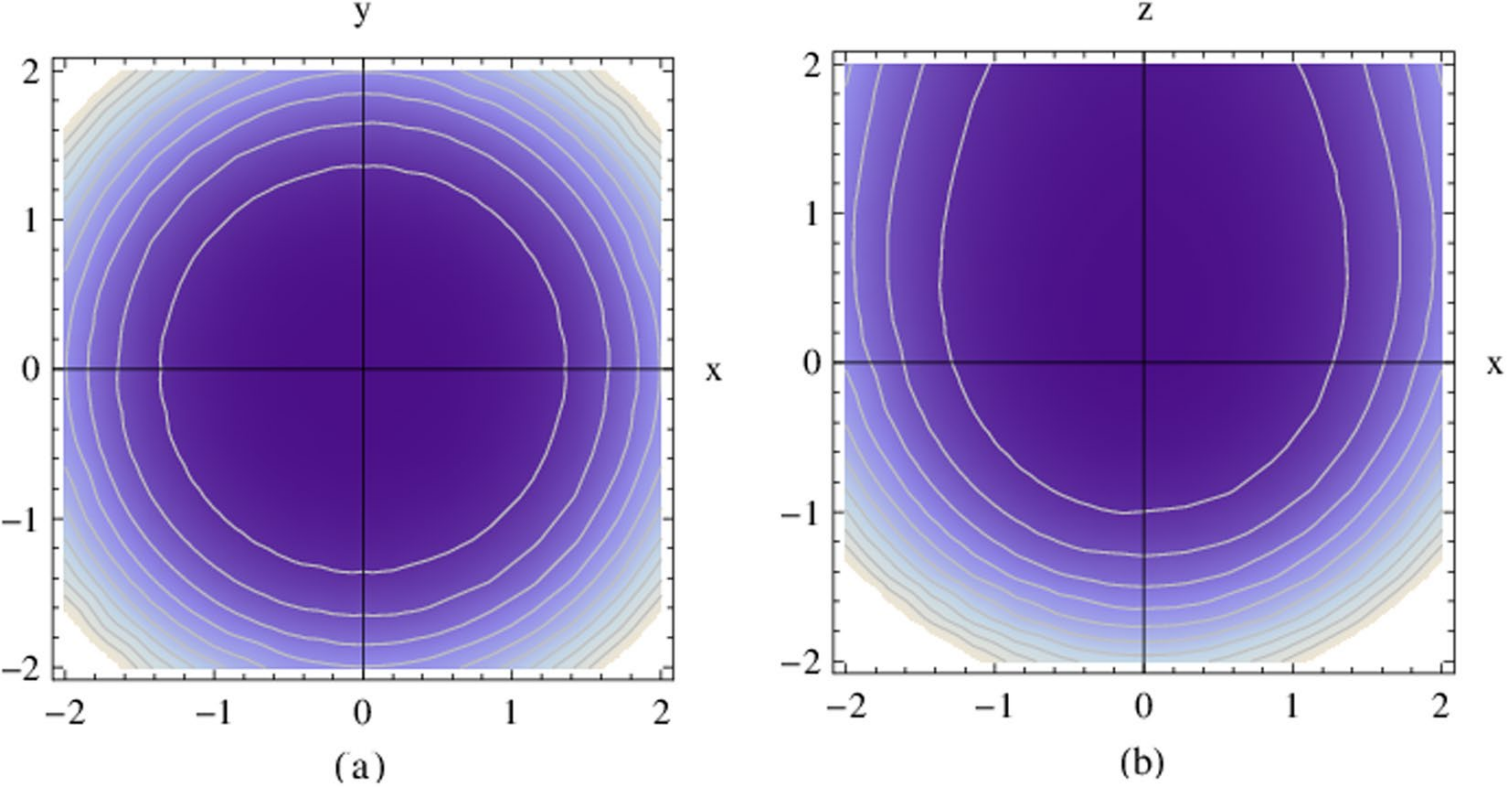
Dependence of the probability density *ρ* in the (**a**) x-y plane and (**b**) x-z plane of the rectangular coordinate system (*x*, *y*, *z*), where *ℏ* = 1, *q* = 1, *n*_*r*_ = 0 and *r*_0_ = 1.

**Figure 2:**
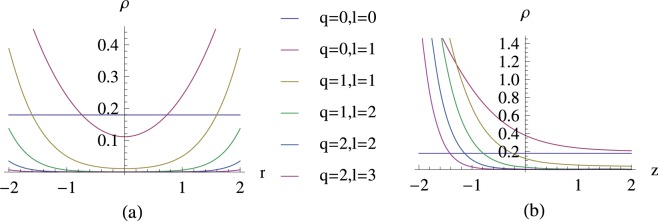
Dependence of the probability density *ρ* for different *q* in the (**a**) x-y plane and (**b**) x-z plane, Where *n*_*r*_ = 0 and *r*_0_ = 1.

**Figure 3:**
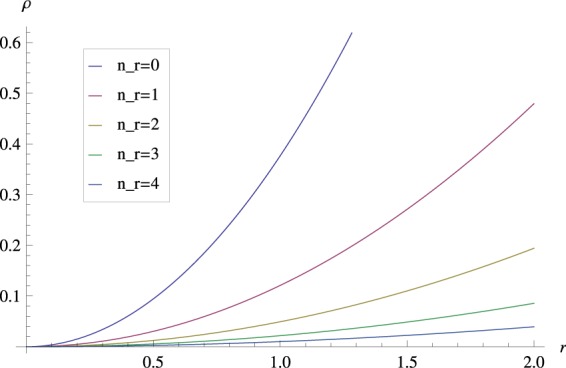
Variations of the probability amplitude *ϕ*_0_ for different radial quantum numbers *n*_*r*_. The other parameters are *q* = 2 and *r*_0_ = 1.

The density distribution of Case 1 is presented. Figure 1 depicts the dependence of the probability density *ρ* in the rectangular coordinate system of (*x*, *y*, *z*). This figure indicates that the probability of charged particle distribution is symmetric around the z-axis. Further, there is a special diffused distribution along the z-axis. Figure 2 depicts the dependence of probability density *ρ* for different *q*, which can be considered to be the physical quantity of the monopole strength. In Fig. 3 the relation between probability density and the radius *r* in different *n*_*r*_ are presented. In this case, the probability density increases with the radius of the *x* − *y* plane, and the probability density is the largest at the bottom of the spherical shell. This distribution properly satisfies the constraint of ▽*ρ*⋅**v** = 0, and the DGE Eq. (23) can be solved.

## Discussion

In this section, the accurate expressions of the nonlinear terms Ω{*ϕ*} in the DGE are considered. Based on the previously obtained density, *R*_*j*_[*ϕ*] can be calculated as follows (in Case 1):

39 $$\begin{array}{c}{R}_{1}={R}_{4}=\mathrm{0;}\,\,\,\,\,\,\,{R}_{2}=\frac{{\mu }^{2}{D}^{2}\mathrm{(5}-3\,\cos \,\theta )}{8{r}^{2}\mathrm{(1}-\,\cos \,\theta )};\\ {R}_{3}=\frac{4{\mu }^{2}{D}^{2}\mathrm{(5}-3\,\cos \,\theta )}{5{r}^{2}\,\sin \,{\theta }^{2}};\,\,\,{R}_{5}=\frac{{\mu }^{2}{D}^{2}\mathrm{(5}-3\,\cos \,\theta )}{16{r}^{2}\mathrm{(1}-\,\cos \,\theta )}\mathrm{.}\end{array}$$

In summary, *R*_1_ = *R*_4_ = 0; ${R}_{2}=2{R}_{5}=\frac{{\mu }^{2}{D}^{2}\mathrm{(5}+3\,\cos \,\theta )}{8{r}^{2}\mathrm{(1}+\,\cos \,\theta )}$ and ${R}_{3}=\frac{{\mu }^{2}{D}^{2}\mathrm{(5}-3\,\cos \,\theta )}{2{r}^{2}\,\sin \,{\theta }^{2}}$. Hence the sum of nonlinear terms Ω{*ϕ*} in Eq. (22) can be given by

40 $${\rm{\Omega }}\{\varphi \}=F^{\prime} {c}_{2}{R}_{2}+F^{\prime} {c}_{3}{R}_{3}+F^{\prime} {c}_{5}{R}_{5},$$

where $F^{\prime} {c}_{2}=\frac{3}{16\mu }\sqrt{\frac{5}{2}}$, *F*′*c*_3_ = 1/8*μ*, and $F^{\prime} {c}_{5}=-\,\frac{1}{16\mu }-\frac{3}{8\mu }\sqrt{\frac{5}{2}}$. *F*′*c*_1_ and *F*′*c*_4_ can take arbitrary real values. The Hamiltonian of Eq. (7) is further rewritten to give a general form of the nonlinear Schrödinger equation that also includes the linear case^25^.

41 $$H={H}_{0}+i\sum _{j=1}^{2}\,{\nu }_{j}{R}_{j}[\varphi ]+\sum _{j=1}^{5}\,{\eta }_{j}{R}_{j}[\varphi ],$$

where *H*_0_ is the linear part of the Hamiltonian with Dirac potential. In the last two terms, *ν*_1_ = − $\frac{1}{2\mu }$, *ν*_2_ = − $\frac{1}{2}F$, *η*_1_ = *F*′*c*_1_, *η*_2_ = − $\frac{1}{4\mu }$ + *F*′*c*_2_, *η*_3_ = $\frac{1}{2\mu }$ + *F*′*c*_3_, *η*_4_ = *F*′*c*_4_, and *η*_5_ = $\frac{1}{8\mu }$  + *F*′*c*_5_. Note that *F* = 0 and *R*_1_ = 0 so that the second term containing imaginary numbers is eliminated. Five independent gauge-invariant quantities that label the classes of equations in the family can be introduced to understand the physical meaning of this set of coefficients. These gauge invariants are provided as nonlinear combinations of the original coefficients^25,36^.

42 $$\begin{array}{c}{\tau }_{1}={\nu }_{2}-\frac{1}{2}{\eta }_{1};\,\,{\tau }_{2}={\nu }_{1}{\eta }_{2}-{\nu }_{2}{\eta }_{1};\,\,{\tau }_{3}=\frac{{\eta }_{3}}{{\nu }_{1}}\\ {\tau }_{4}={\eta }_{4}-{\eta }_{1}\frac{{\eta }_{3}}{{\nu }_{1}};\,\,{\tau }_{5}={\nu }_{1}{\eta }_{5}-{\nu }_{2}{\eta }_{4}+{{\nu }_{2}}^{2}\frac{{\eta }_{3}}{{\nu }_{1}}\mathrm{.}\end{array}$$

The corresponding value of the gauge invariants are ${\tau }_{1}=-\,\frac{1}{2}F^{\prime} {c}_{1}$, ${\tau }_{2}=\frac{1}{8{\mu }^{2}}-\,\frac{3}{32{\mu }^{2}}\sqrt{\frac{5}{2}}$, ${\tau }_{3}=-\,\frac{5}{4}$, ${\tau }_{4}=F^{\prime} {c}_{1}+\frac{5}{4}F^{\prime} {c}_{4}$, and ${\tau }_{5}=-\,\frac{1}{32{\mu }^{2}}+\frac{3}{16{\mu }^{2}}\sqrt{\frac{5}{2}}$. Because the quantities *F*′*c*_1_ and *F*′*c*_4_ take arbitrary values, they can be set to zero to satisfy the condition that results in time-reversal invariance, which indicates that the wave function *ϕ*(**r**, *t*) of DGE satisfies *ϕ*(**r**, *t*) = *ϕ*(**r**, −*t*) if *τ*_1_ = *τ*_4_ = 0. Note that the transformation *t* → −*t* is equivalent to setting ${{\nu }_{j}}^{T}=-\,{\nu }_{j}(j=1,2)$ and ${{\eta }_{j}}^{T}=-\,{\eta }_{j}(j=1,\ldots \mathrm{5)}$, where the superscript *T* denotes time reversal^25^. However, the parameter *τ*_3_ ≠ −1 breaks the Galilean invariance, i.e. the DGE solutions do not satisfy the gauge transformation of *ϕ*′(**r**, *t*) = exp[−*iμ*(**v**⋅**r** + **r**^2^*t*/2)]*ϕ*(**r** + **v***t*, *t*)^32^. Comparing with previous studies^37^, it can be observed that this situation can be mainly attributed to the vorticity of the velocity field in Eq. (5). Furthermore, *τ*_2_ and *τ*_5_ characterize the deviation from linearizability.

## Conclusion

A dissipation quantum system with a Dirac potential was investigated in this study. First, the motions of a particle in rotational superfluid were indicated, and an effective potential was introduced so that the Schrödinger equation would exhibit the same form as that exhibited by the classical fluid equation. After the gauge transformation, a subfamily of DGE containing the Dirac potential was obtained. In particular, the vector potentials *A*_*N*_ in the northern hemisphere and *A*_*S*_ in the southern hemisphere were derived from the velocity fields of Case 1 and Case 2, respectively. After analyzing the exact solutions of the DGE in the selected model, the relevant density distributions were observed to be similar to those of the monopole potential. The dissipation characteristics of the system were discussed for the DGE, thereby describing the dissipative quantum system. It was observed that this dissipative quantum system broke the Galilean invariance although it was time invariant.

The solution in this study can be applied to simulate the distribution of the Dirac potential field in a quantum damped oscillator system. Furthermore, by changing the type of the central potential, it may be possible to extend the solution to other dissipative systems with different Ω{*ϕ*}. In general, if a central potential satisfies *r*^2^*V*_0_(**r**) → 0 and the forms of its relevant wave function conform to *R*_*l*_(**r**) ∝ *r*^*l*^ when *r* → 0, this potential can be constructed as the additional radial potential of the model and the corresponding results will also satisfy the constraint conditions of $\nabla \times {\bf{v}}\ne 0$ and ▽*ρ*⋅**v** = 0. Therefore, in addition to the harmonic oscillator potential, this solution can be extended to other potentials such as the spherical square potential (hard core model).
